# Transparent semipermeable dressings for peripherally inserted central catheters in neonates – should we be concerned?

**DOI:** 10.1186/1753-6561-5-S6-O10

**Published:** 2011-06-29

**Authors:** I Soulake, N Bochaton, C Vassant-Allemoz, G Renzi, R Pfister, J Schrenzel, D Pittet, W Zingg

**Affiliations:** 1 Infection control programme, University of Geneva Hospitals, Geneva, Switzerland; 2 Department of Paediatrics, University of Geneva Hospitals, Geneva, Switzerland; 3 Microbiology Laboratory, University of Geneva Hospitals, Geneva, Switzerland

## Introduction / objectives

Transparent, semi permeable dressings (TSD) have become a standard means of dressing catheter insertion sites in adults, children and infants. TSDs are used for peripherally inserted central venous catheters (PICCs) in neonates although their firm adherence to the catheters prevent dressing change after 7 days.

## Methods

All neonates with a PICC in 2010 were prospectively included in this single-centre observational study. There was no scheduled dressing change for TSD. Non-selective culture plates, pressed to the PICC insertion site upon catheter removal, were incubated for 24 hours and colony counts within a 1 cm radius were compared to the time of dressing. Dressing-time was defined as days TSDs were in place. Standard definitions were used for central line associated bloodstream infections (CLABSI).

## Results

In total, 48 PICCs with a median dwell-time of 8 days (IQR 6-12) totalled 445 catheter-days. Three CLABSI cases (6.7/1000 catheter-days) were detected with a mean time-to-infection of 19 days. No CLABSI case was found before day 15. There was significant correlation between dressing-time and CLABSI (OR 1.16; 95%CI 1.01-1.33; p=0.036) as well as between skin colony counts and dressing-time (p=0.001). While little growth was found in the first days such was significant after 7 days.

## Conclusion

Although TSDs have advantages such as allowing visual inspection of the insertion site, their use for PICCs in neonates should be reconsidered as such dressings cannot be removed easily after 7 days and may stay in place for up to 30 days. This in turn may cause serious infectious complications.

## Disclosure of interest

None declared.

